# Reactive Jumps Preserve Skeletal Muscle Structure, Phenotype, and Myofiber Oxidative Capacity in Bed Rest

**DOI:** 10.3389/fphys.2019.01527

**Published:** 2020-01-15

**Authors:** Dieter Blottner, Maria Hastermann, Robert Weber, Regina Lenz, Guido Gambara, Ulrich Limper, Jörn Rittweger, Alessandra Bosutti, Hans Degens, Michele Salanova

**Affiliations:** ^1^Charité–Universitätsmedizin Berlin, Corporate Member of Freie Universität Berlin, Humboldt-Universität zu Berlin, Berlin Institute of Health, Institute of Integrative Neuroanatomy, Berlin, Germany; ^2^NeuroMuscular Group, Center of Space Medicine and Extreme Environments, Berlin, Germany; ^3^Department of Movement and Neurosciences, German Sports University, Cologne, Germany; ^4^Charité Comprehensive Cancer Center, Berlin, Germany; ^5^German Cancer Consortium (DKTK), German Cancer Research Center (DKFZ), Heidelberg, Germany; ^6^German Aerospace Center (DLR), Institute of Aerospace Medicine, Cologne, Germany; ^7^Department of Anesthesiology and Intensive Care Medicine, Merheim Medical Center, Hospitals of Cologne, University of Witten/Herdecke, Cologne, Germany; ^8^Department of Life Sciences, University of Trieste, Trieste, Italy; ^9^Research Centre for Musculoskeletal Science & Sports Medicine, Manchester Metropolitan University, Manchester, United Kingdom; ^10^Institute of Sport Science and Innovations, Lithuanian Sports University, Kaunas, Lithuania

**Keywords:** bed rest, disuse, muscle atrophy, capillarization, oxidative capacity, countermeasure

## Abstract

Identification of countermeasures able to prevent disuse-induced muscle wasting is crucial to increase performance of crew members during space flight as well as ameliorate patient’s clinical outcome after long immobilization periods. We report on the outcome of short but high-impact reactive jumps (JUMP) as countermeasure during 60 days of 6° head-down tilt (HDT) bed rest on myofiber size, type composition, capillarization, and oxidative capacity in tissue biopsies (pre/post/recovery) from the knee extensor *vastus lateralis* (VL) and deep calf *soleus* (SOL) muscle of 22 healthy male participants (Reactive jumps in a sledge, RSL-study 2015–2016, DLR:envihab, Cologne). Bed rest induced a slow-to-fast myofiber shift (type I –>II) with an increased prevalence of hybrid fibers in SOL after bed rest without jumps (control, CTRL, *p* = 0.016). In SOL, JUMP countermeasure in bed rest prevented both fast and slow myofiber cross-sectional area (CSA) decrements (*p* = 0.005) in CTRL group. In VL, bed rest only induced capillary rarefaction, as reflected by the decrease in local capillary-to-fiber ratio (LCFR) for both type II (pre vs. post/R + 10, *p* = 0.028/0.028) and type I myofibers (pre vs. R + 10, *p* = 0.012), which was not seen in the JUMP group. VO_2__max__Fiber_ (pL × mm^–1^ × min^–1^) calculated from succinate dehydrogenase (SDH)-stained cryosections (OD_660 nm_) showed no significant differences between groups. High-impact jump training in bed rest did not prevent disuse-induced myofiber atrophy in VL, mitigated phenotype transition (type I – >II) in SOL, and attenuated capillary rarefaction in the prime knee extensor VL however with little impact on oxidative capacity changes.

## Introduction

Microgravity-induced changes in the musculoskeletal system of astronauts present potential risks to their safety and health as they may lead to muscle or bone injury particularly during demanding (extravehicular) activity and thus limit their ability to perform routine occupational daily tasks and thus mission success (Adams et al., 2003). Therefore, considerable effort is being undertaken to develop effective multisystem (e.g., neuromusculoskeletal, cardiovascular) and multimodal interventions (e.g., nutrition, aerobic/resistive exercise, plyometrics) (Ploutz-Snyder et al., 2018; Scott et al., 2019) to attenuate the effects of human deconditioning and impaired control of performance following prolonged space missions (Petersen et al., 2016; Lang et al., 2017).

Apart from muscle atrophy (Bodine, 2013), prolonged disuse such as during bed rest (Hargens and Vico, 2016) also induces arterial structural remodeling and decreases in blood flow to lower limb muscles (Arbeille et al., 2008; Thijssen et al., 2011). A reduced blood flow of the mostly inactive muscle in disuse may result in structural capillary rarefaction as mechanical signals and endothelial cell shear stress are crucial for capillary maintenance and angiogenesis (Hudlicka et al., 1992). Besides the delivery of oxygen, an adequate capillary perfusion is important for sufficient nutritional supply and removal of heat and waste products and also for tissue remodeling (Ballak et al., 2016), repair (Omairi et al., 2016), and exercise tolerance (Hirai et al., 2018). Capillarization and myofiber mitochondrial oxidative capacity of muscle fibers and motor units are positively correlated with their fatigue resistance (Degens and Veerkamp, 1994). Thus, in addition to reduced local tissue oxidative capacity, capillary rarefaction occurring in disuse may also lead to (i) lower fatigue resistance (Degens and Alway, 2006; Hendrickse and Degens, 2019), (ii) abnormal intramuscular fat infiltration (Pagano et al., 2018), and (iii) exacerbation of tissue inflammation and damage, as for example observed with chronic obstructive pulmonary disease (COPD) in the hypoactive patient (Abdulai et al., 2018).

Gravitational unloading during spaceflight is associated with both muscle atrophy and reduced oxidative capacity in humans (Baldwin, 1996; Fitts et al., 2013; Milanovic et al., 2015) and rodents (Baldwin, 1996; Shenkman et al., 2002; Milanovic et al., 2015). Countermeasure training in space might be specifically difficult for oxidative capacity, even when it shows effectiveness for structural and glycolytic components of muscle function (Rittweger et al., 2018). Notably, oxidative antigravity muscles (such as *m. soleus*) are more prone to microgravity-induced muscle weakness, reduced fiber cross-sectional area (CSA), and slow-to-fast fiber-type transition (Baldwin, 1996) than non-postural, that is, mixed fast-slow muscle such as the *quadriceps femoris vastus lateralis* (VL) (Degens and Alway, 2006). After 19 days of medium-term bed rest (MTBR), the myofiber oxidative capacity was significantly reduced irrespective of fiber type in both *soleus* (SOL) and VL muscle and accompanied with a reduction in whole-body maximal oxygen uptake (VO_2__max_) (Bosutti et al., 2016). The absence of a significant loss of capillaries resulted in a capillarization in relative excess to oxidative capacity (Bosutti et al., 2016). Whether capillary supply remains in relative excess to oxidative capacity during prolonged disuse and whether capillary rarefaction keeps pace with decrements in fiber size are unknown.

Jumping is a high-intensity, low-volume type of training that does not require much time, yet, reactive jumping induces high strain and strain rates (Milgrom et al., 2003), which have been suggested to be key determinants for bone strength (Turner et al., 1995). In addition, jump training has repeatedly been shown to increase leg muscle strength (Saez-Saez de Villarreal et al., 2010) and leg stiffness (Kramer et al., 2012). Reactive jumping has the potential to prevent both musculoskeletal and cardiovascular deconditioning in disuse with minimal time expenditure and maximal training outcome (Kramer et al., 2017b, 2019; Maggioni et al., 2018). High-intensity interval training with reactive jumps can also be used to preserve lean body mass and counteract cardiovascular deconditioning to a similar extent as traditional high-volume, moderate-intensity aerobic training (Kramer et al., 2017a). Tolerance of reactive jump exercise, training, and functional benefits were shown in healthy male participants in a 60 day long-term head-down tilt (HDT) bed rest study using reactive jumps on a sledge system (RSL Study 2015–2016) solicited by the European Space Agency (ESA) (Kramer et al., 2017a). However, the effects of this high-impact exercise countermeasure on bed rest-induced skeletal muscle atrophy, fiber type transitions, oxidative capacity, and potential capillary rarefaction are not yet known.

To investigate this, we analyzed changes in myofiber CSA and the induction/prevention of myofiber type shift (slow-to-fast) in VL and SOL biopsy samples from the RSL Study using high-resolution confocal microscopy, quantitative structural capillarization parameters, combined with fiber maximal oxygen consumption (VO_2__max_) estimations by optical density (OD_660__nm_)-based semiquantitative mitochondrial succinate dehydrogenase (SDH) histochemistry performed on freshly cut tissue cryosections. Together with previous RSL-Study reports (Kramer et al., 2017b, 2018; Koschate et al., 2018; Maggioni et al., 2018; Schoenrock et al., 2018), the present investigations using muscle biopsy samples from two different functional leg muscles (prime knee extensor and deep plantar flexor) provide evidence that high-impact reactive jumps maintained myofiber size and phenotype with a trend only to also preserve oxidative capacity analyzed in the prime knee extensor VL in HDT bed rest. Nevertheless, high-impact interval training could be a short and effective exercise surrogate for multimodal/multisystem training protocols on Earth and for crew members in long-duration space missions.

## Materials and Methods

### General Study Information

This single-center, parallel-group, crossover design, and randomized controlled training study was sponsored by ESA and conducted at the German Aerospace Center (DLR:envihab human physiology facility) in Cologne. Reactive jumps in a sledge jump (RSL) system was used as countermeasure during a 6° HDT bed rest study with *n* = 24 healthy male participants enrolled and randomly assigned to control [CTRL, *n* = 12, aged 30.0 ± 5.76 years, body mass index (BMI) 23.5 ± 2.2] or an RSL intervention group (JUMP, *n* = 12, aged 29.9 ± 6.6 years, BMI 23.4 ± 1.67) (Kramer et al., 2017b). One participant of the CTRL group discontinued the study 4 days before bed rest (BDC-4) for medical reasons unrelated to the study resulting in *n* = 11 final CTRL. Other RSL study information, anthropometric data, participant inclusion/exclusion criteria, diet, medical and paramedical treatments are reported elsewhere (Kramer et al., 2017b). The Northern Rhine Medical Association (Ärztekammer Nordrhein) in Düsseldorf, Germany, as well as the Federal Office for Radiation Protection (Bundesamt für Strahlenschutz) Ethics Committee approved this study in accordance with the World Medical Association Code of Ethics, Declaration of Helsinki (1964). All subjects gave their informed written consent prior to the participation in this study and were aware that they could withdraw from the experiment at any time. This study was registered with the German Clinical Trial Registry (#DRKS00012946, September 18, 2017).

### Study Design and Exercise

The present study design consisted of 15 days of baseline data collection (BDC-15 through BDC-1), 60 days of strict HDT bed rest in supine position (24/7 HDT1 through HDT60), and 15 days of recovery (R + 0 through R + 14) and was conducted in two campaigns (first campaign, August 28–November 27, 2015; second campaign, January 26–April 26, 2016). During the adaptation and recovery phases (BDC and R), physical activity was restricted to free movement within the ward and re-education training tailored to each participant’s status during recovery. The sledge jump system (SJS) was developed by Novotec Medical GmbH (Pforzheim, Germany). Study participants were familiarized with the correct jumping technique in the SJS system in 9 × 30 min sessions during BDC. The JUMP group underwent prescribed RSL training (48 training sessions in total during 60 days of RSL) on an HDT SJS at supine body position using shoulder straps for presetting of different body and leg pressing forces measured by two separated force plates (each foot). Jump forces are generated by two low-pressure cylinders (450 N full capacity). Briefly, familiarization was achieved during BDC-7 by the following setup: nine 30 min sessions, warm-up, ankle joint mobilization, six deep static squats, six heel raises, three submaximal countermovement jumps, one series of submaximal hops, and three submaximal countermovement jumps, followed by one series of 10 hops each). Preset jump forces started with 50% body weight (b.wt.) gradually increasing to 100% b.wt. Four different training sessions (A–D) were prescribed providing 80–90% average body load, different hop bouts (2 × 12 to 4 × 15) and amounts of countermovement jumps (10 × 3 to 1 × 12) at different durations (from 90 s, 3 or 4 min). During exercises, verbal encouragement and visual feedback were provided to participants on an SJS feedback monitor. More details about the training duration, intervals, and loading forces are described elsewhere (Kramer et al., 2017a, b).

### Muscle Biopsy

Muscle biopsies (50–80 mg) from the lateral thigh (VL) and deep calf (SOL) were obtained at BDC-5 (*pre*), HDT-59 (*post*), and R + 10 (*rec*) under local anesthesia (2 ml 1% Lidocaine) using a Rongeur forceps (Blottner et al., 2014).

### Immunohistochemistry

#### Reagents

Primary antibodies used: monoclonal antibodies specific for slow myosin heavy chain (sMyHC, MHC-I) isoform (mouse IgG1, M8421, dilution 1: 1,000) and fast MyHC (fMyHC, MyHC-IIa and -IIx) isoforms (mouse IgG1, M4276, dilution 1:2,000) (Sigma, St. Louis, MO, United States) to identify the two predominantly expressed type I and type IIa myosin isoforms in human muscle (Andersen, 2003) an established and fast protocol to check for putative myosin isoform transition patterns in limited amounts of high-quality cryosections from human biopsy (Rudnick et al., 2004); polyclonal antibody specific for dystrophin (rabbit polyclonal, SC-15376, dilution 1:100) (Santa Cruz Biotechnology, Inc., Santa Cruz, CA, United States); mouse monoclonal CD31-antibody (Dako M0823), platelet endothelial cell adhesion molecule-1 (PECAM-1, dilution 1:50). Secondary antibodies: Alexa 488-conjugated, Alexa 555-conjugated, Alexa 635-conjugated affinity-purified goat anti-mouse and anti-rabbit (Invitrogen Corp., diluted 1:4,000, Carlsbad, CA, United States). Reagents: For multiple immunostaining, mouse-on-mouse (MOM) IgG blocking reagent from Vector Laboratories, Inc. (Burlingame, CA, United States); alkaline phosphatase-conjugated secondary antibodies were visualized with nitro blue tetrazolium chloride (NBT)/5-bromo-4-chloro-3-indolyl phosphate (BCIP) alkaline substrate solution buffer from Pierce (Thermo Fisher Scientific LSR, Rockford, IL, United States). All other chemicals were of analytical and molecular grade.

### Immunostaining of Myofibers

Human skeletal muscle biopsy cryosections [LEICA CM-2800, 8 microns (μm) thickness] from VL and SOL pre- and post-BR groups (CTRL *n* = 11, JUMP *n* = 12) were either double- or triple-immunolabeled with anti-slow/-fast MyHC for myofiber type I and type II, respectively, combined with anti-dystrophin (membrane immunomarker). To balance intersubject variability, subject-matched (post- vs. pre-BR) cryosections from CTRL and JUMP groups were always incubated in parallel with the same immunohistochemical protocol. Cell nuclei were counterstained with blue DAPI (Vectashield, Vector). Images of immunostained specimen were acquired by confocal microscopy (TCS SP-2 and SP-8, LEICA Microsystems). Quantification (myofiber size and phenotype distribution) was performed with LEICA Confocal Software 2.7. For capillary analysis, an epifluorescence microscope (Axioplan; Zeiss, Oberkochen, Germany) equipped with a Cool Snap digital camera (Visitron Systems GmbH, Puchheim, Germany) was utilized. Digitized images were processed with MetaVue software (Meta Series 7.5.6; system ID: 33693; Molecular Devices, Sunnyvale, CA, United States).

### Myofiber CSA and Fiber-Type Composition

Immunofluorescence images were analyzed as described previously (Salanova et al., 2009). For each muscle biopsy, the numerical and areal fiber-type composition was calculated (Barnouin et al., 2017). Mean CSA (μm^2^) of type I, type II, and hybrid myofibers coexpressing both slow and fast MyHC immunomarkers (type I/II hybrids) was measured in digitized images of MyHC and dystrophin triple-immunostained cryosections by use of the Leica 2.61.1537 image analysis software. Blinded analysis was performed on a total number of 13.013 (SOL) and 15.013 (VL) individual myofibers (mean number of myofibers analyzed per biopsy: 242 VL, 197 SOL). Analyses were made in areas of transversely cut myofibers on cryosections where capillary locations were represented by single image points (pixels) around single myofiber profiles.

### Analysis of Muscle Capillarization

Muscle capillarization was determined in the jump-related knee extensor VL only. PECAM-1-immunostained cryosections were processed by BTablet (BaLoH Software^1^) and further analyzed by AnaTis (BaLoH Software^1^) software. Briefly, AnaTis calculates capillary domains and parameters related to muscle fiber size and composition (Bosutti et al., 2015). A capillary domain is the area of tissue surrounding a capillary delineated by equidistant boundaries from adjacent capillaries (Hoofd et al., 1985). The capillary domain appears to give a good reflection of the oxygen supply area of a capillary (Al-Shammari et al., 2012; Bosutti et al., 2015). Data for capillary density (CD), capillary-to-fiber ratio (C:F), and local capillary-to-fiber ratio (LCFR) were obtained, reflecting the number of capillaries supplying a fiber, and was given by the sum of the domain fractions overlapping that fiber (Bosutti et al., 2015). Note that the LCFR of a fiber takes into account remote capillaries, thus allowing the determination of the capillary supply to a fiber even when it lacks direct capillary contacts (Bosutti et al., 2016). The capillary fiber density (CFD) was calculated as the LCFR divided by the myofiber CSA and was expressed as the number of capillaries per square millimeter. To get information about the capillary contacts per fiber, reflecting the oxygen exchange area per fiber (Hepple, 1998), the LCFR per fiber perimeter (LCFR-perimeter ratio) was also calculated. The method of capillary domain (Voronoi polygon) also provides information about the distribution of capillaries within the tissue and allows one to calculate the capillary supply to fibers that lack direct capillary contacts (Degens et al., 1992; Egginton et al., 2001; Wüst et al., 2009; Bosutti et al., 2015).

### SDH and Maximal Oxygen Consumption

The optical density (OD) of the mitochondrial SDH histochemical stain on cryosections is a measure of the mass-specific maximal oxygen consumption of a fiber and was analyzed on freshly prepared biopsy cryosections (van der Laarse et al., 1989; Wüst et al., 2009; Bosutti et al., 2015). Briefly, an 8 μm thick serially cut section adjacent to the PECAM capillary-immunostained section was incubated at 37°C in the dark for 45 min in 37 mM sodium phosphate buffer pH 7.6 with 74 mM sodium succinate and 0.4 mM tetra-nitroblue-tetrazolium. The reaction was stopped with 0.01 N HCl (5 s) with water. Wet sections were mounted in glycerol gelatin (van der Laarse et al., 1989; Bosutti et al., 2015). Photomicrographs of stained cross sections were then captured, and the OD_SDH_ of a fiber was determined by measuring the absorbance of the final reaction product (precipitate) using an interference filter at 660 nm (van der Laarse et al., 1989; Bosutti et al., 2015). Absorbance was converted to OD by a calibration curve specific for each individual section created with a set of filters with known OD (ImageJ software) to minimize bias related to differences in lighting and precipitation rates. The SDH activity is calculated as the staining rate: increase of absorbance units at 660 nm per micrometer section thickness per second of incubation time (ΔA_660_ μm^–1^ s^–1^_max_ = 6,000^∗^OD_660_^∗^thickness in μm^–1*^incubation time in s^–1^). The OD_SDH_ analysis was performed at the same time in the same incubation for all muscle biopsies (cryosection) obtained from each participant. VO_2max_ of muscle fibers was calculated from these data as described previously (Bosutti et al., 2015; Barnouin et al., 2017).

### Statistical Analyses

Data were tested for normal distribution by Kolmogorov-Smirnov Test. CSA/myofiber type composition: myofiber-specific CSA values (μm^2^) are given as means and standard deviation (SD) (see Supplementary Table S1) and myofiber type composition is presented as bar chart with means and error bar with 2 SEM (standard error of mean, Supplementary Table S1). Changes in CSA and myofiber type composition depending on the time point (pre/post/R + 10) and training (JUMP vs. CTRL) were statistically evaluated by two-way repeated-measures analysis of covariance (RANCOVA) adjusted for pre time point and campaign, with Bonferroni correction for multiple comparisons. Within-group comparisons between time points (pre/post/R + 10) were analyzed by one-way repeated-measures analysis of variance (RANOVA) and Bonferroni correction for multiple comparisons. Capillarization: non-parametric Friedman test with *post hoc* Dunn-Bonferroni testing was performed to analyze changes in CD (mm^2^), CFD, DAF, C:F, LCFR; LCFR per fiber perimeter, capillary number, SDH, and VO_2__max_Fiber. From each group (JUMP, CTRL), six regular samples (with longitudinally oriented myofiber bundles) were analyzed. In all tests, a value of *p* < 0.05 was accepted as statistically significant. All statistical analyses were performed using SPSS (v.25, IBM, Armonk, NY, United States).

### Data Availability

Study raw data are found in Supplementary Tables S1, S2 and available from the authors upon request. The data are to be made available in the Erasmus Experiment Archive^2^.

## Results

### Myofiber Type CSA and Myofiber Type Composition

**FIGURE 1:**
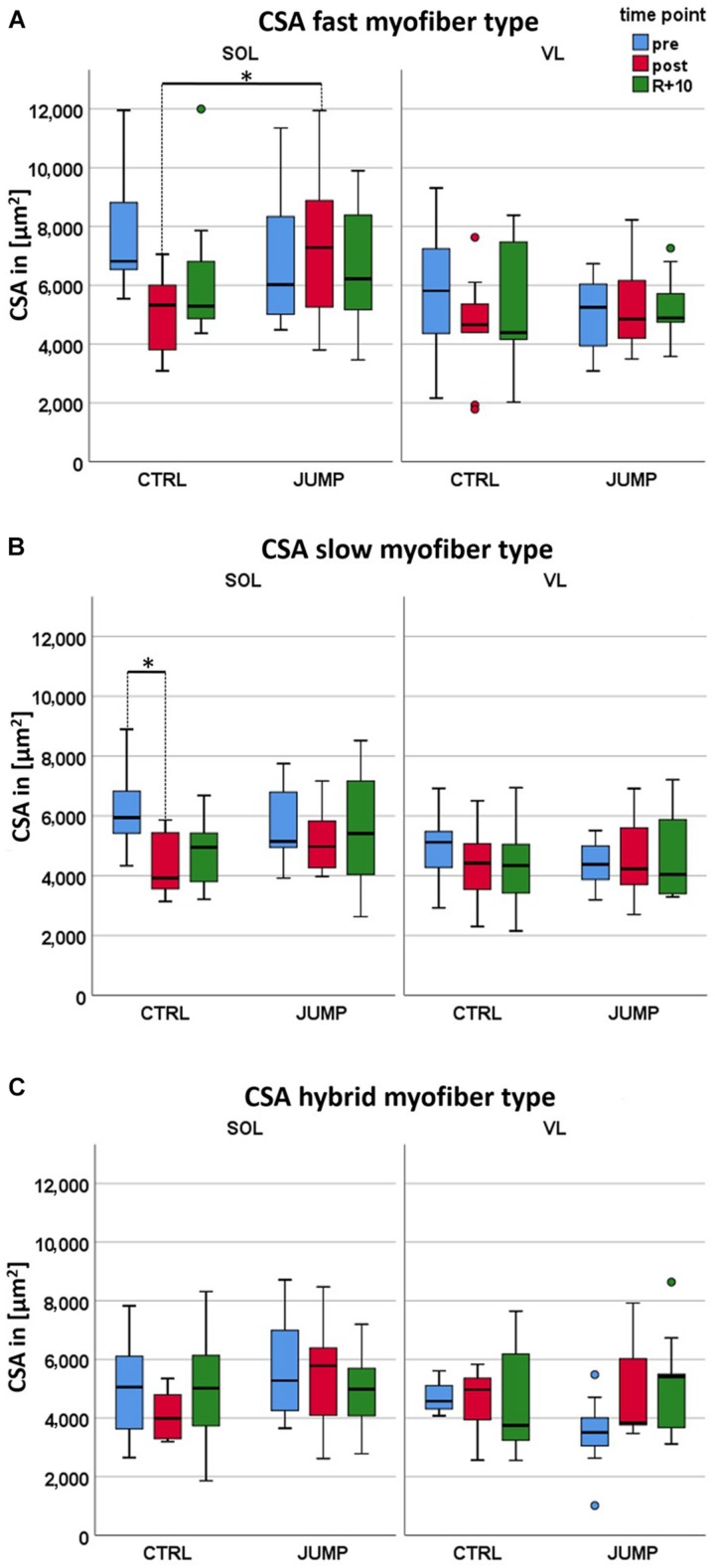
Effect of 60 days RSL study bed rest on soleus (SOL) and vastus lateralis (VL) myofiber cross-sectional area (CSA) with and without JUMP training as countermeasure at three different time points (pre/post/recovery). **(A)** Box plots showing fast myofiber II mean CSA (μm^2^) of control (CTRL) (SOL *n* = 10, VL *n* = 9) and JUMP (SOL *n* = 12, VL *n* = 12). **(B)** Box plots showing slow myofiber I mean CSA (μm^2^) of CTRL (SOL *n* = 10, VL *n* = 9) and JUMP (SOL *n* = 12, VL *n* = 12). CSA values denote pre bed rest before (pre, blue), at end (post, red), and at 10 days of recovery after bed rest (R + 10, green). **(C)** Box plots showing fast (blue) and slow MyHCs (red) vs. hybrids (expressing both MyHCs, green) mean CSA (μm^2^) of CTRL (SOL *n* = 6, VL *n* = 4) and JUMP (SOL *n* = 11, VL *n* = 8). ^∗^Significance *p* < 0.0001.

Differences in fast SOL myofiber CSA were found in CTRL vs. JUMP (*p* = 0.001) not seen in VL at any time point (Figure 1A and Supplementary Table S1). Variable differences were found in both pre and post SOL and VL CSA in either group at pre and post time points (e.g., SOL, CTRL: 6,000–12,000 μm^2^; SOL, JUMP (2,000–9,000 μm^2^) due to subject variability in each randomized group. Differences in slow SOL myofibers were only found in CTRL (*p* = 0.05), however, not seen in JUMP (Figure 1B). In the VL, however, no significant bed rest-induced atrophy was observed (Figure 1A and Supplementary Table S1). The SOL data show a statistically significant interaction between time and group in the CSA fast myofiber type (*p* = 0.035). *Post hoc* comparison showed that CSA fast myofibers differ between JUMP and CTRL groups at the post time point (*p* = 0.001, Supplementary Table S1). Such an interaction indicates that the changes over time (in supine bed rest) differed between CTRL and JUMP. Myofiber size changes in hybrid fibers (expressing both MyHC-I and II simultaneously) showed a trend only (Figure 1C and Supplementary Table S1). No significant changes were found in either muscle at R + 10 time point of recovery (Figure 1).

**FIGURE 2:**
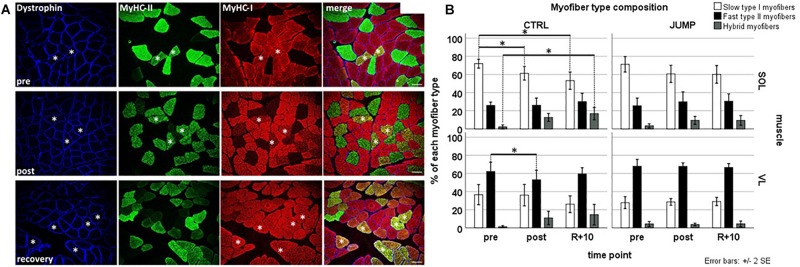
Representative subject-matched/paired images of myofiber type composition (slow/fast/hybrid) in RSL biopsies from one bed rest participant. **(A)** Triple immunostained cross sections [soleus (SOL)] with anti-dystrophin (blue) anti-type II (green) anti-type I (red) MyHC before (pre; upper panel), at end of head-down tilt (HDT) BR (post; middle panel), and recovery (R + 10, lower panel). Some hybrid myofibers (yellow immunostain, coexpressing both type I and type II MyHCs) are marked with white asterisks. Bar = 75 μm. **(B)** Quantification (bar graph) of myofiber phenotype composition (percent of total) between groups and time points. Upper panel (SOL), lower panel [vastus lateralis (VL)]; open bars (left column) control (CTRL) group, closed bar (right column) JUMP group. Percentage of slow type I, fast type II, and hybrid myofibers (expressing both markers) in SOL and VL muscle of BR subjects without (CTRL, type I/II SOL *n* = 10, VL *n* = 9, hybrid SOL *n* = 6, VL *n* = 4) and with exercise (JUMP, type I/II SOL *n* = 12, VL *n* = 12, hybrid SOL *n* = 11, VL *n* = 8) as countermeasure. A pre > post/recovery decrease in CTRL SOL type I myofibers (*p* = 0.001/0.001), pre > post decrease in CTRL VL type I myofibers (*p* = 0.037), and simultaneous increase in CTRL SOL hybrids (pre < rec *p* = 0.016) was observed. No changes were found in SOL and VL muscles from JUMP. ^∗^Significance at *p* < 0.05, SPSS GLM with *post hoc* Bonferroni correction, bar graph/box plots (means) with median ± 2 SE.

Immunohistochemistry analyses (Figures 2A,B) showed that both CTRL and JUMP groups’ *pre* SOL muscle biopsies consisted of predominantly slow type I myofibers (CTRL 75.62% and JUMP 70.13%, respectively) and fewer fast type II myofibers (CTRL 23.06% and JUMP 26.83% respectively). As expected, the *pre* muscle biopsies of the VL showed an inverse pattern with predominantly fast myofibers (II:I, CTRL 63.66%:35.80%, and JUMP 67.76%:28.66%). Subject-matched analysis (Figure 2B) of post muscle biopsies from CTRL revealed a significant change in myofiber type composition in SOL muscle in CTRL group, where type I myofibers decreased from pre to post (*p* = 0.001) as well as pre to recovery (*p* = 0.001). The proportion of hybrid myofibers increased at the same time from pre to recovery (*p* = 0.016). The relative number of hybrid myofibers increased from 1.33% pre to 12.63% post in CTRL SOL and from 0.55% pre to 10.92% post in CTRL VL, whereas the relative number of type I myofibers decreased from 75.62% pre to 61.3% post in CTRL SOL (Figure 2B). There was no change observed in the myofiber type composition of JUMP group in either muscle (Figures 2A,B), indicating close-to-normal myofiber patterns with JUMP in bed rest. The VL of CTRL group revealed a significant reduction of type II myofibers from pre to post (*p* = 0.037) time points. JUMP SOL showed a trend toward a reduction of slow myofibers between pre and post time points only (*p*-values see Supplementary Table S1 and Figure 2B). Myofiber type changes (in%) in SOL type II were similar between JUMP and CTRL groups, however, due to higher intersubject variability of the JUMP group, the change was not significant.

### Muscle CD, CFD, and C:F

**FIGURE 3:**
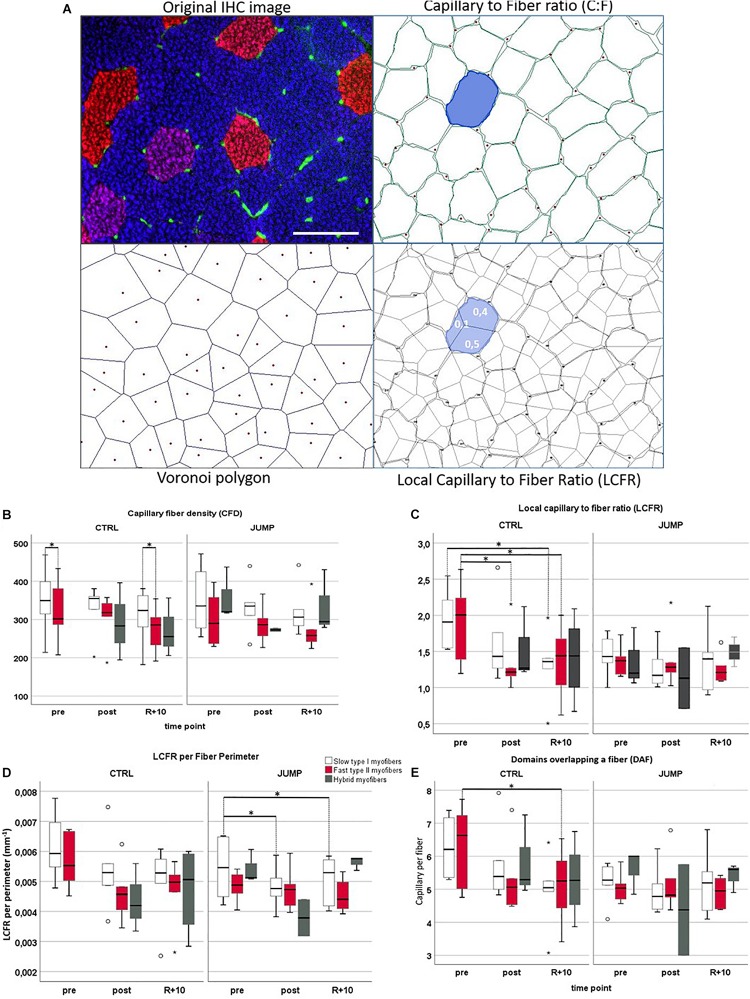
Capillarization in vastus lateralis (VL) muscle from RSL study groups at three different time points (pre/post/R + 10). **(A)** Representative VL cryosection [head-down tilt (HDT) + 58, upper left] immunostained with slow MyHC-I (red), fast MyHC-II (blue), and platelet endothelial cell adhesion molecule 1 (PECAM-1, green), identifying capillary structures. Upper right shows automatically drawn myofiber borders (green) and capillaries (red dots) using BTablet Software (BaLoH). Lower left shows calculated Voronoi polygons (VP, also referred to as capillary domains; gray lines) representing the area of tissue closer to a given capillary (red dots) than neighboring capillaries. Lower right shows overlap of VP with myofiber borders, local capillary-to-fiber ratio (LCFR) illustrated in blue with fraction size supplied through different capillaries indicated as numbers (0.1, 0.4). Scale bar (top left): 100 μm. **(B–E)** Box plots for different capillarity parameters: **(B)** capillary fiber density (CFD), **(C)** local capillary-to-fiber ratio (LCFR), **(D)** LCFR per fiber perimeter, and **(E)** domains overlapping a fiber (DAF) in different myofibers (slow = no color/fast = red/hybrids = gray), groups (CTLR vs. JUMP), and time points (pre/post/R + 10). ^∗^Significant differences *p* < 0.05; non-parametric Friedman with *post hoc* Dunn-Bonferroni correction, box plots (means) with median ± 2 SE; small circles (o) = statistical outliers.

Figure 3A shows a representative set of images (original immunohistochemistry vs. modeled cartoons) used for quantitative parameter determination of muscle capillarity. No significant CD (mm^2^) and CFD changes between the three time points (pre/post/R + 10) or between groups could be detected in VL muscle (Supplementary Table S2 and Figure 3B). However, contrary to previous findings (Wüst et al., 2009), CFD values differed significantly between myofiber types in VL muscle: CTRL pre slow > fast (*p* = 0.028), recovery slow > fast (*p* = 0.043) (Figure 3C). No C:F changes of VL muscle (Figures 3A,B) were found between training groups (CTRL vs. JUMP) or different time points (pre/post/R + 10).

### LCFR

In CTRL, LCFR reduced significantly between fast type VL myofibers pre vs. post (*p* = 0.028) and pre vs. recovery R + 10 (*p* = 0.028) and furthermore reduced in slow type myofibers pre vs. R + 10 (*p* = 0.012). No changes in LCFR were seen in JUMP (Supplementary Table S2 and Figures 3A,B). This may be explained by the coincidental presence of hybrid fibers particularly seen in post and R + 10 time points.

### LCFR per Fiber Perimeter

The LCFR parameter reflects the number of capillaries supplying a fiber and is given by the sum of the domain fractions overlapping that fiber. In JUMP group, LCFR per fiber perimeter reduced significantly between slow type VL myofibers pre vs. post (*p* = 0.028) and pre vs. recovery R + 10 (*p* = 0.028). In CTRL, LCFR per perimeter showed no significant changes (Figure 3D).

### Domains Overlapping a Fiber (DAF)

The DAF parameter provides information about the distribution of capillaries within the tissue and allows for calculations of the capillary supply to fibers that lack direct capillary contacts. In CTRL, fast myofibers showed significantly reduced DAF parameter values when comparing pre to R + 10 time points (*p* = 0.028) (Figure 3E).

### Oxygen Demand/Capacity-VO_2_ max_*Fiber*_ (L × kg^–1^ × min^–1^)

**FIGURE 4:**
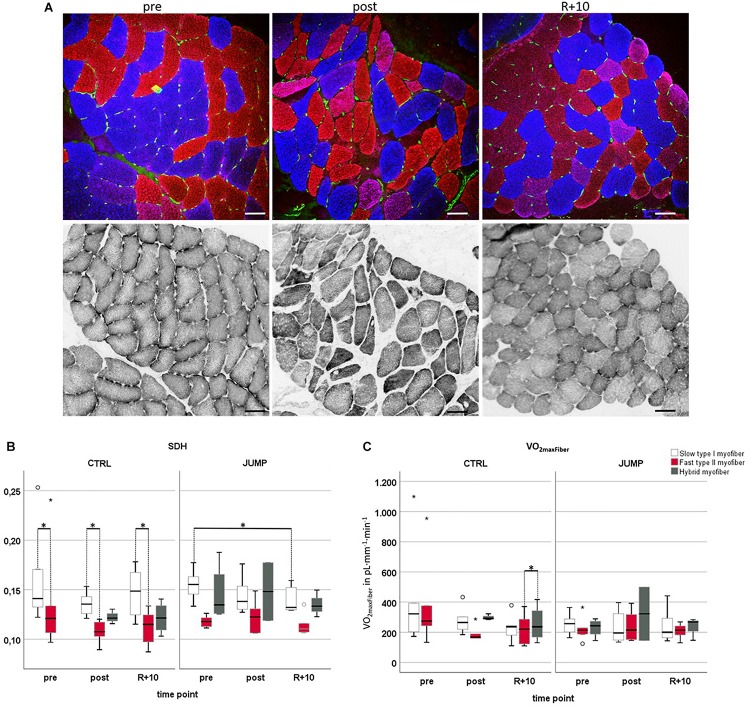
Oxidative capacity analyzed by semiquantitative histochemical succinate dehydrogenase optical density analysis (OD_SDH_) using RSL study biopsy cryosections. **(A)** Representative subject-matched/paired microscopic images at three different time points [vastus lateralis (VL), pre/post/R + 10]. Upper panel, immunostaining for slow MyHC-I (red), fast MyHC-II (blue), and capillary platelet endothelial cell adhesion molecule 1 (PECAM-1; green). Lower panel, adjacent cryosections with matched area stained for SDH histochemical activity at 37°C, index of mitochondrial activity (gray values). Scale bars = 75 μm. **(B)** Determination of optical density of SDH marker at 660 nm (OD_660_) in control (CTRL) (myofiber type I/II *n* = 6, hybrid pre *n* = 0 post *n* = 3 R + 10 *n* = 4) or exercise (JUMP, VL myofiber type I/II *n* = 6, hybrid pre *n* = 4, post *n* = 2, R + 10 = 3). The specific SDH activity (OD_SDH_) was always higher in CTRL pre/post/recovery (R + 10) type I vs. type II myofibers (pre *p* = 0.028, post *p* = 0.043, recovery *p* = 0.014). In the JUMP group, the amount of SDH activity was significantly different (reduced) in slow myofibers at R + 10 vs. pre (*p* = 0.028). **(C)** VO_2maxFiber_ values showed a significant difference between recovery fast < hybrid (*p* = 0.014) with no changes in JUMP. CTRL group (left, myofiber type I/II *n* = 6, hybrid pre *n* = 0, post *n* = 3, R + 10 *n* = 4), JUMP group (right, myofiber type I/II *n* = 6, hybrid pre *n* = 4, post n = 2, R + 10 = 3). SDH and VO_2__*maxFiber*_ of slow type 1 (red), fast type 2 (blue), and hybrid myofibers (green) in participants without (CTRL) and with exercise (JUMP) at pre/post/recovery time points of head-down tilt (HDT) bed rest. ^∗^Significance at *p* < 0.05, box plots (means) with median ± 2 SE.

Figure 4A presents a set of images from subject-matched and adjacent cryosections (slow/fast/PECAM triple immunohistrochemistry vs. SDH histochemistry) from one bed rest participant for documentation. Histochemical SDH (OD_SDH_) activity of muscle fibers (Figures 4A,B) was quantified to investigate to what extent long-term HDT bed rest and JUMP intervention might have affected fiber oxidative capacity (Figure 4). As an index of the aerobic capacity/mitochondrial content of muscle fibers, we determined the OD_SDH_ histochemical precipitate at 660 nm (OD_660_) since OD_660_ is linearly related to fiber maximal oxygen demand (MO_2__max_) (Wüst et al., 2009; Bosutti et al., 2015). OD_SDH_ was higher in CTRL pre, post, and recovery slow type I myofibers compared to fast type II myofibers (pre *p* = 0.028, post *p* = 0.043, recovery *p* = 0.014). In the JUMP group, OD_SDH_ was significantly reduced in slow myofibers I between pre and R + 10 time points (*p* = 0.028). VO_2_ max_F__iber_ values showed a significant difference between recovery fast < hybrid (*p* = 0.014) in CTRL, and no changes in the JUMP group (Figure 4C).

## Discussion

### Major Findings and Study Outcome

The present biopsy analyses of the SOL and VL muscles generally demonstrated the reactive jump training-based positive anti-deconditioning outcome (Kramer et al., 2017a, b, 2018) after 60 days of HDT bed rest on tissue/myofiber-scale level. At end of bed rest, preservation of histomorphological myofiber size and phenotype composition in the jump-related hip and calf muscles was found particularly in JUMP. The present tissue scale (biopsy) outcomes from the RSL long-term bed rest suggest that apart from size and phenotype preservation by exercise in bed rest, muscle capillarization and oxidative capacity showed only little changes at least in knee extensor VL by short but high-impact JUMP countermeasure interventions under extended disuse conditions. The present outcome supports the notion that short duration and high-impact jumps could at least partly serve as an alternative surrogate to other multimodal exercise protocols, such as combined neuromuscular, musculoskeletal, as well as cardiovascular interventions, in long periods of disuse on Earth and perhaps also in spaceflight. This might be noteworthy especially to possible applications in microgravity environments where crew time constraints exist, but countermeasures often have extensive duration and limited output not least due to low compliance and adherence to prescribed training sessions (Ploutz-Snyder et al., 2018).

### Structural Changes at Tissue/Cell Scale

Following RSL Study bed rest in the CTRL group, we observed a marked decrease in myofiber type I and type II CSA in the deep calf SOL as previously reported (Blottner et al., 2006; Salanova et al., 2014). Notably, although there was some trend visible for recovery in SOL type I myofiber size following 10 days of re-ambulation, the fiber size outcome was still below baseline (*pre* bed rest). Myofiber size enlargement in both types of myofibers in R + 10 VL muscle was not observed; however, type I VL myofibers showed a trend toward decreasing mean CSA value from pre > post > R + 10 from both CTRL/JUMP groups. In a previous bed rest study with resistive vibration exercise (RVE), atrophy changes in myofibers were in fact documented only in the CTRL group in both slow and fast SOL myofibers, whereas myofiber size in VL muscle was maintained, however even in the absence of RVE exercise training (Salanova et al., 2015). The lack of myofiber CSA change in disused VL with bed rest may be explained by still incompletely understood bed rest-only effects, for example, due to muscle-specific blood perfusion rates in SOL vs. VL (at 6° HDT leg position), differential pressing forces and their outcome at soft tissue/leg muscle anatomical regions (SOL vs. VL, supine leg), or even by differential metabolic state in a mixed fast-type VL vs. mainly slow-type SOL (Irimia et al., 2017; Rudwill et al., 2018). From human leg musculature, the postural SOL is more prone to unloading than VL following bed rest immobilization (Belavy et al., 2009).

### Myofiber Type Composition (Hybrid Formation)

Myosin type I and IIa are the two predominant myosins in normal human VL with up to 30% increase in hybrids following resistance training in bed rest (Andersen, 2003). Muscle unloading leads to a myofiber phenotype shift (type I > type II) with an augmented number of hybrid myofibers accompanying the process (Borina et al., 2010; Blottner et al., 2014). We reproduced these findings partly in the CTRL group of the present RSL Study in both muscles (elevated hybrid fiber levels are potential signs of increased tissue remodeling) and found a significant reduction in the amount of slow type I myofibers in SOL of the CTRL group pre vs. post/recovery. A previous study showed that bed rest-only may not always induce full shifts in myosin phenotype particularly in human VL biopsies (Bamman et al., 1998), supporting the notion of elevated hybrid fiber formation as sign for residual myofiber populations expressing both slow/fast myosins as also found in the present study. However, maximal force resistance training (flywheel) during an 84 days bed rest study mitigated myofiber CSA loss and myosin shifts in the VL comparable to the obvious lack of changes seen after reactive jumps in the present RSL study (Gallagher et al., 2005). The mode of countermeasure (high-impact reactive jumps) used in the RSL study was equally effective, at least in VL, in mitigating myofiber type shift (slow to fast) induced through bed rest than another short and highly efficacious countermeasure protocol in HDT bed rest based on a vibration-augmented resistive exercise (RVE) protocol (Salanova et al., 2014).

### Capillarization

Muscle capillarization/aerobic capacity is linked to exercise and myofiber phenotype distribution in athletes in sports (Pringle et al., 2003; Prior et al., 2003). However, this obvious correlation between capillarity and muscle phenotype or mass is less well-reflected in chronic bed rest disuse with exercise as countermeasure. In line with a previous 90 days HDT BR study (Rudnick et al., 2004) and other medium duration bed rest (MDBR) studies of 42–45 days (Ferretti et al., 1997; Krainski et al., 2014), no changes in capillarity (C:F, LCFR, CFD) could be found in the CTRL groups’ VL pre vs. post except for a significant reduction in CTRL VL myofiber type I LCFR after BR (pre vs. post/rec). This result however is critical as *pre* LCFR values for both CTRL myofiber types are markedly higher than those of the JUMP group. Of note, however, is that while resistance training (Rudnick et al., 2004) and endurance training (ET) (Andersen and Henriksson, 1977; Ingjer, 1979; Hoppeler et al., 1985; Jensen et al., 2004; Schmutz et al., 2010; Busso and Fluck, 2013; Egan and Zierath, 2013) lead to a significant increase in C:F ratio, thus providing (transient) enhancement in capillarization in VL, reactive jumps as countermeasure in the present study did not lead to changes in C:F or any other measure parameters of capillarization in otherwise mainly resting conditions in bed rest. The reasons for differential muscle capillarity in bed rest observed in the present RSL study are likely due to many other bed rest effects related to disuse-induced muscle signaling, microvascular growth and adaptation, and variable tissue mechanical stress parameters (see below).

### Oxidative Capacity–Oxygen Consumption

In contrast to previous findings (Bosutti et al., 2016) that showed immobilization-induced loss in oxidative capacity in both fast/slow myofibers, we observed a significantly higher oxidative capacity in type I myofibers as compared to type II myofibers. The present RSL Study findings however support the notion of a reduced mitochondrial density/mitochondrial enzyme activity (i.e., mitochondria-associated SDH activity) due to bed rest as proposed earlier (Ferretti et al., 1997). The present investigation on biopsy tissue scale is in obvious line with another RSL report on systemic heart-rate (HR)-based pulmonary vs. muscular oxygen (O_2_) uptake kinetics that also showed little changes in bed rest independent of countermeasure intervention (Koschate et al., 2018).

### Muscle Capillarization, Tissue Viscoelasticity, and Molecular Mechanisms

While correlations for example between muscle capillarization and tissue viscoelasticity (based on perfusion with blood and lymphatic fluids) are but hypothetical to this end, one could also ask about the possibility for the extracellular matrix (ECM) in skeletal muscle, which is sometimes generally referred to as “fascia” (Zugel et al., 2018). Accumulating evidence suggests that ECM not only matters in terms of mechanics but also takes part in determining the skeletal muscle’s phenotype and probably also takes some part in strength output. As previously reported from the same study, rectus femoris muscle (medial part of quadriceps femoris) resting muscle stiffness (viscoelasticity) determined by the non-invasive Myoton technology increased in JUMP during RSL long-term bed rest and returned to baseline (pre bed rest) faster than observed in CTRL following recovery thereafter (Schoenrock et al., 2018). At bed rest end, for example, rectus femoris in CTRL showed a reduction in resonance frequency and material stiffness, which can jointly be thought to represent passive muscle “tone” (Schoenrock et al., 2018). Possible links may also exist between structural and functional myofiber properties, tissue viscoelasticity, capillarization, and microvascular network adaptation in disuse without and with exercise that need further investigation. Notably, the still incompletely understood microvascular network tissue changes during bed rest disuse in human skeletal muscle apparently seem to undergo time-dependent adaptation mechanisms (short- vs. long-term), for example, by intramuscular vascular signaling mechanisms, such as proposed for nitric oxide (NO) (Percival et al., 2010) controlled by mechanotransduction and mechanical loading as, for example, reflected by earlier studies investigating effects of short-duration, high-intensity exercises (high-intensity intermittent training (HIT), such as sprint and strength training, where consistent adaptation in capillarity was also not found (Tesch et al., 1984; Lüthi et al., 1986; Hoier et al., 2013; Gliemann, 2016; MacInnis and Gibala, 2017). With repetitive exercise, the consequent elevation in microvascular shear stress stimulates in conjunction with abluminal mechanical factors, morphogenic adaptations, and proliferation of endothelial cells in muscle capillaries (Bassett and Howley, 2000; Gustafsson and Kraus, 2001; Prior et al., 2003; Valdivieso et al., 2017), which may explain similar mechanisms to occur in bed rest exercise protocols. A limited number of studies in animals and humans have determined the presence of angiogenic factors in skeletal muscle at the gene and protein level in association with acute exercise and training (Breen et al., 1996; Lloyd et al., 2003; Rullman et al., 2007). Among the pro-angiogenic factors, for instance, NO, generated by endothelial NO synthase enzyme (eNOS, NOS-2), plays an important role in the maintenance of endothelial homeostasis (Heiss et al., 2015). Similar to VEGF, eNOS is upregulated by shear stress (Williams et al., 2006a), and NO has been shown to regulate VEGF expression (Tsurumi et al., 1997). Other pro-angiogenic factors, such as matrix metalloproteinases (MMPs) and angiopoietin-2, which are important for the degradation of the extracellular matrix and capillary destabilization during sprouting angiogenesis (Rivilis et al., 2002; Hoier et al., 2012), certainly need to be further investigated in future bed rest analog studies. Interestingly, it was shown that with elevated training intensity, the release of some anti-angiogenic (angiostatic) factors increases at mRNA level (Hoier et al., 2013), suggesting tight regulation of capillary growth with muscle activity (Placanica et al., 1999; Williams et al., 2006b; Egginton, 2009; Malek and Olfert, 2009).

### Countermeasure Effectiveness

Results from the RSL study on tissue scale (muscle biopsy) presented in this work confirmed that the SJS only partly affected oxidative capacity in VL (trend only) but preserved myofiber CSA and phenotype transition in SOL. Our findings support previous work on leg power, muscle peak force, and surface electromyographic (EMG) measurements with plyometrics (jump training) in bed rest from the same RSL study (Kramer et al., 2017a). So far, the only other study that had achieved preservation of muscle fiber size and function was the Berlin Bed Rest study, which had used resistive exercise in combination with superimposed whole-body vibration (Blottner et al., 2006). To achieve such full maintenance of the musculature during bed rest is more challenging than one would think, in particular in the calf. For example, a training regimen based on the flywheel, which is highly effective to generate muscle hypertrophy under ambulatory conditions failed to prevent muscle atrophy in bed rest (Alkner and Tesch, 2004; Rittweger et al., 2005). One explanation could be that plyometric exercises, that is, the consecutive stretch and shortening of the contracting muscle, involve an important stimulus for muscle growth. Indeed, bone strains, which reflect the regional musculoskeletal forces (Yang et al., 2015), are greatest during plyometric exercises, such as hopping and jumping. Trampoline-based plyometrics is able to increase mass and power in the VL muscle in both young and older populations (Franchi et al., 2019). Notably, stretch-shortening cycles occur not only in reactive jumping but also during whole-body vibration (Cochrane et al., 2009). Therefore, provision of stretch-shortening cycles, either *via* reactive jumping or *via* vibration, could be a smart route for countermeasure exercise in microgravity (Gruber et al., 2019) and in reduced gravity (Weber et al., 2019). To what extent high-impact exercise modalities like SJS protocols with reactive jumps and other forms of hops can be translated to real clinical settings (e.g., muscle disorders, rehabilitation) or microgravity conditions (e.g., inflight countermeasure protocols on the International Space Station) for better astronaut’s health management during space missions and reconditioning thereafter is currently discussed (Weber et al., 2019).

## Conclusion

Bed rest induced atrophy, as indicated by a set of intramuscular tissue-/cell-scale parameters, such as reduced myofiber CSA, altered slow/fast myofiber type shift (type I > II), can be prevented by a training regimen of short-duration and high-impact reactive jumps as countermeasure in healthy male participants during a long-term HDT bed rest period of 60 days. However, only a trend could be determined in JUMP for maintenance in capillarity and oxidative capacity otherwise challenged by chronic disuse in HDT bed rest. This may reflect some lack of efficacy of the countermeasure chosen in this study on the one hand, but it could also be due to the principal difficulty of maintaining muscle perfusion while exercising in a supine position in bed rest or in microgravity. However, 10 days after bed rest deconditioning, histomorphological signs of recovery in leg skeletal muscle fibers were found particularly in the JUMP group, suggesting compliance with improved reconditioning thereafter. Considering the brevity of the performed training protocol in comparison to the robust intramuscular structural tissue-/cell-scale preservation, reactive jumps may serve as an exercise surrogate to other existing multimodal/multisystem protocols in rehabilitation, clinical settings, as well as in spaceflight.

## Data Availability Statement

The datasets generated for this study are available on request to the corresponding author.

## Ethics Statement

The Northern Rhine Medical Association (Ärztekammer Nordrhein) in Düsseldorf, Germany, as well as the Federal Office for Radiation Protection (Bundesamt für Strahlenschutz) Ethics Committee approved this study in accordance with the World Medical Association Code of Ethics, Declaration of Helsinki (1964). All Subjects gave their informed written consent prior to the participation in this study and were aware that they could withdraw from the experiment at any time.

## Author Contributions

DB contributed to the study and design, data analysis, and drafted and wrote the manuscript. RW, RL, and GG contributed to the raw data collection (immunostaining, morphometry, and confocal images) and quantitative data analysis. AB and HD contributed to the methodology and data analysis (capillarization and oxidative demand). MH contributed to the data analysis (statistics), graphs and tables, and manuscript drafting. UL and JR performed the muscle biopsies. MS wrote the project, conceived the experiments, and contributed to the study design, coordination, and manuscript drafting. All authors read and approved the final version of the manuscript.

## Conflict of Interest

The authors declare that the research was conducted in the absence of any commercial or financial relationships that could be construed as a potential conflict of interest.

## Footnotes

1 http://www.baloh.nl

2 http://eea.spaceflight.esa.int/portal/

## Supplementary Material

TABLE S1 Raw data CSA SOL/VL, percent Myotype SOL/VL. Two-way repeated measures analysis of covariance (RANCOVA) adjusted for pre time point and campaign, with Bonferroni correction for multiple comparisons. Within group comparisons between time points (pre/post/R + 10) were analyzed by One-way Repeated Measures Analysis of Variance (RANOVA). Bonferroni correction for multiple comparisons was applied where necessary. *P*-value^∗^ = one way RANOVA test within subjects effects (Greenhouse-Geisser), *p*-value group = test between subject effects, *p*-value group^∗^time = test within subjects effects (Greenhouse-Geisser), *p*-value *post hoc* = if *p*-values for group and group^∗^time were significant *post hoc* analysis was performed including Bonferroni correction for multiple comparisons.

TABLE S2 Raw data VL capillarization. Non-parametric Friedman test with *post hoc* Dunn–Bonferroni testing was performed, pairwise comparison by Wilcoxon/Mann–Whitney *U*-test in cases of missing values. *P*-value fiber type – comparison between different myofiber types within a group, *p*-value group – comparison between groups at the same timepoint, *p*-value time – comparison within one group between different time points.
